# Standardization of the antibody-dependent respiratory burst assay with human neutrophils and *Plasmodium falciparum* malaria

**DOI:** 10.1038/srep14081

**Published:** 2015-09-16

**Authors:** David Llewellyn, Kazutoyo Miura, Michael P. Fay, Andrew R. Williams, Linda M. Murungi, Jianguo Shi, Susanne H. Hodgson, Alexander D. Douglas, Faith H. Osier, Rick M. Fairhurst, Mahamadou Diakite, Richard J. Pleass, Carole A. Long, Simon J. Draper

**Affiliations:** 1 The Jenner Institute, University of Oxford, Old Road Campus Research Building, Oxford, OX3 7DQ, UK; 2 Laboratory of Malaria and Vector Research, National Institute of Allergy and Infectious Diseases, National Institutes of Health, Rockville, Maryland, 20852, USA; 3 Biostatistics Research Branch, National Institute of Allergy and Infectious Diseases, National Institutes of Health, Bethesda, USA; 4 KEMRI Centre for Geographic Medicine Research, Coast, P.O. Box 230-80108, Kilifi, Kenya; 5 Liverpool School of Tropical Medicine, Pembroke Place, Liverpool, L3 5QA, UK; 6 Malaria Research and Training Centre, Faculty of Medicine, Pharmacy and Odonto-stomatology, University of Bamako, Bamako, Mali

## Abstract

The assessment of naturally-acquired and vaccine-induced immunity to blood-stage *Plasmodium falciparum* malaria is of long-standing interest. However, the field has suffered from a paucity of *in vitro* assays that reproducibly measure the anti-parasitic activity induced by antibodies in conjunction with immune cells. Here we optimize the antibody-dependent respiratory burst (ADRB) assay, which assesses the ability of antibodies to activate the release of reactive oxygen species from human neutrophils in response to *P. falciparum* blood-stage parasites. We focus particularly on assay parameters affecting serum preparation and concentration, and importantly assess reproducibility. Our standardized protocol involves testing each serum sample in singlicate with three independent neutrophil donors, and indexing responses against a standard positive control of pooled hyper-immune Kenyan sera. The protocol can be used to quickly screen large cohorts of samples from individuals enrolled in immuno-epidemiological studies or clinical vaccine trials, and requires only 6 μL of serum per sample. Using a cohort of 86 samples, we show that malaria-exposed individuals induce higher ADRB activity than malaria-naïve individuals. The development of the ADRB assay complements the use of cell-independent assays in blood-stage malaria, such as the assay of growth inhibitory activity, and provides an important standardized cell-based assay in the field.

The development of efficacious vaccines against major global diseases promises to be one of the most cost effective strategies for achieving significant reductions in global health burden and realizing the possibility of eradication^1^. In the case of *Plasmodium falciparum* malaria, over 200 million people are infected each year leading to approximately 0.6 million deaths^2^,^3^. However despite this burden of disease, the immunological mechanisms which confer protection *in vivo* in humans remain highly debated and poorly understood^4^, and thus vaccine development strategies often suffer from a lack of informed immunological guidance.

Sustained interest in vaccines against the blood-stage of malaria infection has demanded assessment of antibody function against merozoite and infected red blood cell (iRBC) expressed antigens. While it is largely accepted that parasite antigens expressed on the surface of the iRBC are particularly important for naturally-acquired immunity (NAI)^4^, differential expression profiles, and high levels of polymorphism in RBC surface expressed genes between different parasite strains, mean that the majority of blood-stage vaccine efforts have not focused on these targets. Instead merozoite proteins, in particular those involved in the erythrocyte invasion process, have been the focus of vaccine development efforts^5^,^6^,^7^. To this end, the assay of growth inhibitory activity (GIA) – one that assesses anti-merozoite antibodies’ ability to block parasite invasion into the human erythrocyte and/or parasite growth inside the erythrocyte, has been used to direct many blood-stage vaccine development efforts. While the assay of GIA seeks to measure one important mechanism by which vaccine-induced antibodies can block parasite proliferation (essentially cell-independent antibody neutralization), such a mechanism remains to be formally associated with protection following human vaccination^8^ and even then, would likely represent a ‘non-natural’ form of immunity given the relatively poor association between GIA and clinical outcome in the context of studies of NAI^9^.

Consequently, there is significant interest in assays that can guide the development of vaccines that may afford antibody-mediated protection via alternative mechanisms to GIA, and which may help researchers to better understand mechanisms of natural malaria immunity. For example a number of protocols for conducting phagocytosis assays have been described assessing the ability of immune sera to initiate monocyte or neutrophil phagocytosis of either merozoites^10^,^11^ or iRBCs^12^,^13^,^14^. More recently, assays assessing the contribution of antibody-mediated complement-dependent (Ab-C’) inhibition of merozoite invasion have also been reported^15^. In addition, the antibody-dependent cellular inhibition (ADCI) assay, in which monocytes are the effectors of antibody Fc-dependent signaling and subsequent anti-malarial cellular activity has been described^16^. Polyclonal antibodies isolated from the serum of immune African volunteers have been shown to elicit ADCI activity *in vitro*, and these same antibodies were reported to control malaria infection when passively transferred into non-immune humans^17^. However, despite the potential utility of this assay, historically it has proved problematic with regard to demonstrating reproducibility and development of a standardized protocol for use by the malaria community, and thus the assay has been largely under-explored as a vaccine antigen screening tool. More recent work seeking to address this problem, may in time facilitate wider uptake of this approach^18^.

The antibody-dependent respiratory burst (ADRB) assay has also been described^19^,^20^ but, like the ADCI assay, lacks a formal qualification, thus limiting its immediate utility as a vaccine screening or NAI investigative tool. The ADRB assay assesses the ability of anti-merozoite antibodies to activate neutrophils/polymorphonuclear cells (PMNs) via Fc receptors (FcR) to release reactive oxygen species (ROS), a mechanism distinct from assays which assess antibody-induced neutrophil phagocytosis of either merozoites or iRBCs^13^,^21^,^22^,^23^. Although we have recently reported that the ADRB assay does not associate with protection against *P. yoelii* rodent malaria^24^, ADRB activity has been strongly associated with a reduction in *P. falciparum* clinical disease in naturally-exposed individuals in Senegal^19^ lending support to the utility of a reproducible, standardized protocol for use by the malaria research community. In fact, the production of ROS is known to be effective in attenuating growth of intracellular parasites^25^,^26^,^27^ including *P. falciparum*^28^,^29^. This is further supported in mouse models incapable of producing superoxide which experience accelerated malaria parasite multiplication rates^30^. ADRB activity is thus a plausible mediator of protection against *P. falciparum* malaria, supporting the reported association with clinical protection^19^.

Given the reported association between ADRB activity and clinical disease, a reliable protocol for the assay would allow it to be used more broadly in pre-clinical and clinical vaccinology as well as epidemiological assessment of NAI. The assay has three major components: *P. falciparum* merozoites, human PMNs, and human serum. We define optimal parameters for each of these components. In addition we assess both intra- and inter-assay reproducibility in order to define a protocol for testing serum samples for ADRB activity. Using the protocol we develop, we show that a cohort can be quickly and efficiently characterized. We thus provide a standardised protocol for conducting the ADRB assay with human PMNs so that the assay can be used by other laboratories for malaria vaccine development and the evaluation of NAI.

## Results

### Basic Assay Parameter Setup

#### Effector cell number and purity

**Figure 1:**
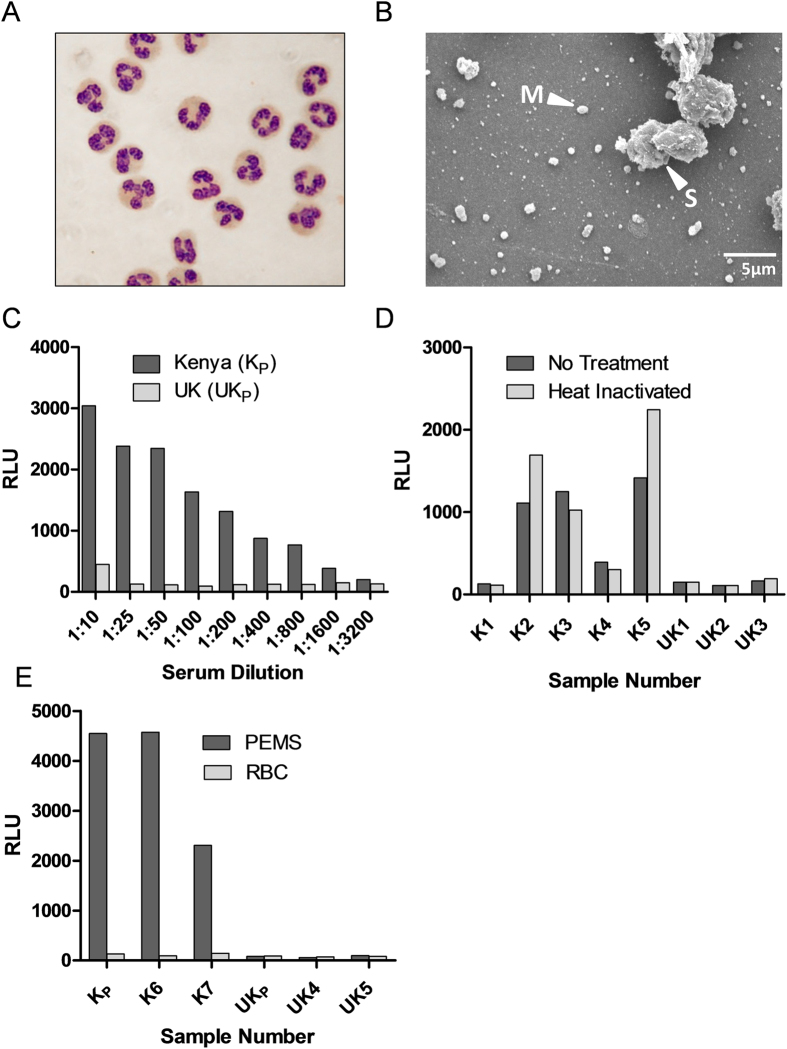
Assessment of ADRB assay parameters. Assay parameters were determined using a plate coated with 18.5 × 10^5^ schizonts/mL and serum from malaria-naïve UK volunteers and Kenyan adults. (**A**) PMNs were isolated from whole blood and confirmed to be >75% pure by Giemsa stained slide. 100 μL of cell suspension was added to each well of the assay at 1 × 10^7^ PMNs/mL. (**B**) Scanning electron micrograph of PEMS preparation showing both merozoites (M) and schizonts (S). (**C**) ADRB activity induced by pooled serum from Kenyan (K_p_) and UK adults (UK_p_) was tested at different serum dilutions and is reported as maximum RLU. (**D**) ADRB activity induced by sera (5 Kenyan and 3 UK individual sera) diluted 1:50 before and after heat inactivation. (**E**) ADRB activity induced by a pool of sera from Kenyan adults and two individual Kenyan volunteers, and a pool of UK, malaria-naïve adults and two individual UK volunteers was assessed using plates coated with either PEMS (dark) or uninfected RBCs (light). Bars represent the mean of duplicate wells tested with the same PMN donor.

Initially PMNs were prepared from whole blood from healthy UK adult donors as described in Methods. The number and purity of freshly isolated PMNs were assessed before addition to the assay in order to meet basic quality control standards. Immediately following the completion of the PMN isolation protocol, cell preparations were examined by Giemsa stained slides (Fig. 1A) and cellular purity determined as percentage PMNs. A cell preparation of >95% PMNs was typical from counting 1000 total cells (data not shown). Typical contaminants were RBCs and monocytes. If ≥25% cells were RBCs, the RBC lysis step was repeated. If ≥25% cells were monocytes, the cell preparation was discarded. A preparation of >75% PMNs was considered sufficient to conduct the assay. After adjusting for PMN purity in the cell preparation, the assay was conducted with 100 μL cell suspension in each well at 1 × 10^7^ PMN/mL in accordance with previous versions of the assay^19^,^24^. Cells were normally used in the assay within 5 minutes of suspension in PMN buffer and no later than 30 minutes; and within 2 hours (maximum 4 hours) of phlebotomy. Typically, enough cells for a complete 96-well assay plate could be isolated from 25 mL whole blood.

#### P. falciparum Parasitophorous vacuolar membrane-Enclosed Merozoite Structures (PEMS) number and purity

The assay was conducted with *P. falciparum* PEMS isolated as described in Methods. PEMS were counted as schizonts before rupturing and suspended in PBS at 18.5 × 10^5^ schizonts/mL – the concentration used in the assay. PEMS were then frozen to rupture the parasitophorous vacuole and release the merozoites and stored at −20 ^°^C for up to 1 year before use. Aliquots were thawed once, the day prior to the assay being conducted, and 100 μL coated onto each well of the assay plate(s) which were then left overnight at room temperature. During assay development, the PEMS suspension used to coat plates was confirmed to contain free merozoites by scanning electron microscopy (SEM) (Fig. 1B), while no such structures were visible in a non-infected RBC preparation (data not shown).

#### Assay readout: Maximum RLU

Luminescence was measured from each well of the 96-well assay plate for 1000 ms every 2 min for 1 h (Supplementary Figure 1) and from the resulting data the following parameters were calculated: maximum RLU; area under the curve (0–60 min); area under the curve (4–20 min); and average RLU (0–30 min). The analyses described below were comparable between all outputs (data not shown), however for consistency with the limited number of previous studies related to this assay^19^,^24^, it was decided to progress using maximum RLU as the assay output (referred to as RLU throughout the rest of the text).

### Effect of serum parameters on ADRB activity

Given the ADRB assay aims to assess the activity of human antibodies against *P. falciparum* blood-stage parasites, it was important to assess how different concentrations and preparations of test serum would affect ADRB activity. Respiratory burst activity induced by a pool of hyper-immune Kenyan sera (tested in duplicate) decreased with increasing serum dilutions, whilst a pool of sera from malaria-naïve UK adults failed to induce ADRB activity at dilutions of 1:25 or more (Fig. 1C). Given this result, it was decided to conduct all future assays with serum diluted 1:50. This was sufficient to observe high level responses from ADRB positive serum without using large volumes of serum (2 μL required per well).

Using the defined serum dilution of 1:50, we also assessed whether the ADRB activity was induced independent of complement. The results showed that there was no significant difference between respiratory burst induction from serum subjected to heat-inactivation compared to non-heat-inactivated samples (Wilcoxon sign rank test: *P* = 0.74; Fig. 1D). In a related series of experiments using a malaria protein-based (as opposed to PEMS-based) assay (Supplementary Methods)^24^, we also showed that there was no difference in induction of ADRB activity from plasma and serum taken from the same volunteers previously immunized with a candidate malaria vaccine targeting the *P. falciparum* merozoite surface protein 1 (PfMSP1) antigen (Supplementary Figure 2)^31^; suggesting that the presence of clotting factors or lithium heparin anticoagulant does not affect the assay output. Using the same assay setup, we also showed that ADRB activity was induced differently by epitope-matched chimeric anti-PfMSP1 monoclonal antibodies of different human isotypes^32^,^33^, such that generally IgA > IgG3 > IgG1 (Supplementary Figure 3).

### ADRB activity is dependent on *P. falciparum* PEMS

Having established that malaria hyper-immune sera from Kenyan adults induced ADRB activity, whilst sera from malaria-naïve UK adults did not, it was important to establish that this activity was specific to the malaria parasite and not a potential reaction to RBCs (in this case isolated from healthy UK adults). Both uninfected RBC and PEMS from malaria iRBC cultures were thus produced and 100 μL of either suspension coated onto plates at 18.5 × 10^5^ schizonts (or RBC)/mL. Upon coating plates with PEMS, serum from Kenyan adult volunteers (with significant levels of prior malaria exposure) elicited ADRB activity. This activity was not induced when coating a plate with uninfected RBC (Fig. 1E). In addition, no ADRB activity was elicited by sera collected from malaria-naïve UK volunteers against either PEMS-coated or RBC-coated plates.

### Reproducibility

**Figure 2:**
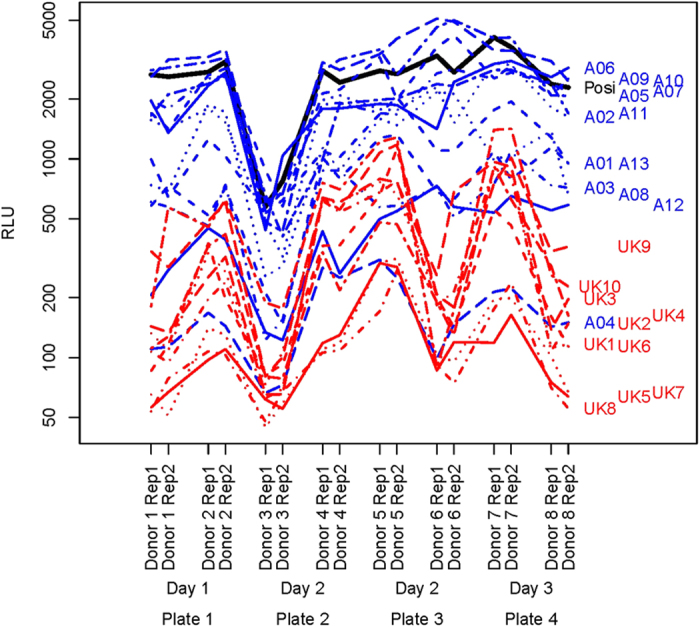
Raw ADRB assay data from repeats with multiple PMN donors. ADRB activity against PEMS was determined for 13 malaria-endemic African (blue) and 10 malaria-naïve UK (red) serum or plasma samples using 8 different neutrophil donors. Samples were diluted 1:50, randomised on PEMS coated plates and were all measured in duplicate. Black = positive hyper-immune pool.

Reproducibility in the ADRB assay was assessed using a small cohort of serum or plasma from 13 African adults and serum from 10 malaria-naïve UK adults. All samples were assayed in duplicate such that with the 23 test samples and the positive control (see below), the whole cohort could be run in 48 wells (half a plate). This layout was replicated on each plate with PMNs from two different healthy UK adult donors and assayed in parallel. This experimental setup was run on three consecutive days, with two plates being run on one day, and thus each sample was run with eight independent PMN preparations (Fig. 2). As above, all sera and plasma were diluted 1:50 and not heat-inactivated, 100 μL PMNs were added at 1 × 10^7^ PMNs/mL, and PEMS were coated onto plates at 18.5 × 10^5^ schizonts/mL.

### Intra-assay reproducibility

**Figure 3:**
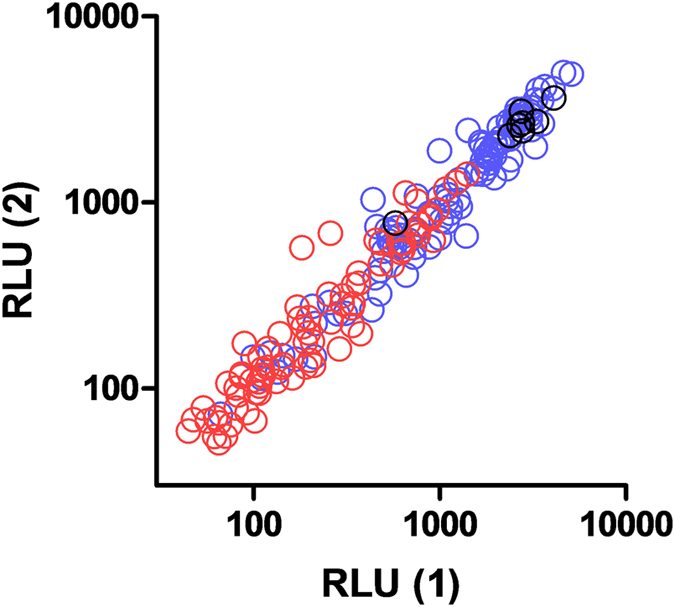
Relationship between intra-assay replicates. ADRB activity against PEMS was determined for 13 malaria-endemic African and 10 malaria-naïve UK serum or plasma samples using 8 different neutrophil donors (*n* = 184) in duplicate. Duplicate values for maximum RLU achieved by each sample in each assay were plotted against each other. Blue = African samples, red = UK sample, black = positive hyper-immune pool.

For each serum sample tested with each PMN donor (*n* = 184) duplicate readings were compared to each other (Fig. 3). Interclass correlation coefficient (ICC) analysis^34^ showed that measuring activity by maximum RLU was highly repeatable under the same conditions (ICC = 0.973). Given this very strong correlation, we asked whether it was necessary to test samples in duplicate as, for the purposes of high throughput of samples, it would be beneficial to only have to test samples in singlicate upon an individual assay plate.

To address the singlicate versus duplicate question, we compared R^2^ values from linear models with sample included only 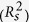, using either singlicates 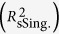 or duplicates 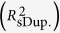. Ideally, we wanted 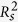 to be close to 1, because we wanted the only variability in our responses to be due to differences in the samples, and we wanted no variability due to differences in PMN donors, plates, day, or replicate. Comparing 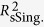 and 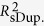 enabled us to see how much the variability due to replicates can be reduced, and thus the 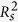 be maximised (there will however, still be unavoidable variability due to PMN donors present). For duplicates, we used the average of the two replicates as a response, so there will be less total variability in the responses compared to using a single replicate as the response. Thus, the proportion of that total variability explained by the sample in the model (i.e. 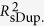) will necessarily be larger than that proportion for the single replicate, 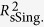. The important question was how much improvement does using duplicates give? To answer this we used the percent increase:


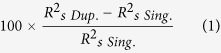


This analysis showed that the % increase in *R*^*2*^_*s*_ due to testing samples in duplicate was less than 7% for all data when assessing African samples only whether analyzed as total values (RLU), indexed values (RLU_p_) (defined below), or log transformed data. When UK samples were included in the analysis, measuring samples in singlicate reduced *R*^*2*^_*s*_ by less than 4% compared to *R*^*2*^_*s*_ when measuring samples in duplicate (Table 1), thus confirming the assay could be acceptably performed in singlicate.

### Inter-assay reproducibility

**Figure 4:**
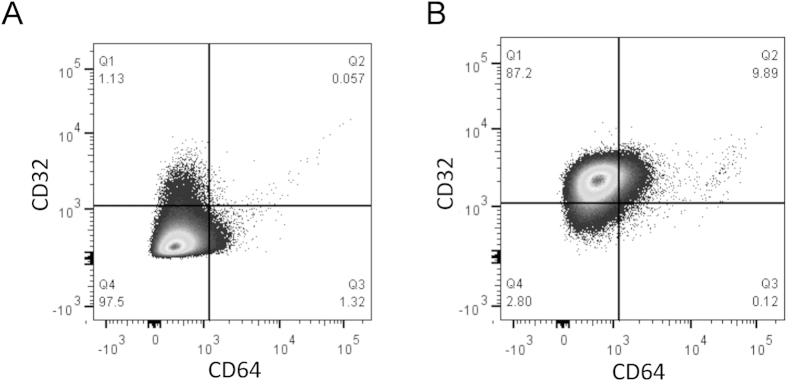
Assessment of FcR on human PMNs. PMNs were isolated from healthy UK volunteers and characterized by flow cytometry with regard to their CD32 and CD64 expression levels on CD16^+^ granulocytes (Supplementary Figure 4). Representative plots from individuals who exhibited either **(A)** CD32^lo^ or (**B**) CD32^hi^ phenotypes.

The raw assay readout in terms of maximum RLU varied significantly by neutrophil donor. The effects of donor are significant even after accounting for day or plate effects (Friedman test: *P* < 0.001, see also Fig. 2). As we needed to prepare fresh human neutrophils each day, there is no way to measure actual day-to-day variation other than as the effect of PMN donor. We thus focused on donor effects for the analysis of inter-assay variability. Using a permutation test we could find no significant plate effects after accounting for donor effects (*P* = 1.00). Notably, during the assay development we had observed that the CD16^+^ granulocyte populations (Supplementary Figure 4) of five healthy UK adults expressed either low (Fig. 4A) or high (Fig. 4B) levels of CD32, which may have contributed to the inter-donor variation we observed.

Potential strategies for indexing data to compensate for PMN donor variation were thus investigated using the cohort of 13 African and 10 UK samples. In accordance with previous iterations of this assay in both mice^24^ and humans^19^, a positive control sample was defined and included on each assay plate against which test samples, under exactly the same conditions, were indexed (RLU_p_) such that:


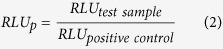


This positive control consisted of a pool of hyper-immune Kenyan adult sera with sufficient volume to run over 1000 plates. Similar to non-indexed data, intra-assay reproducibility for RLU_p_ was high (ICC = 0.926) and the assay could be acceptably run with singlicates (Table 1). We also calculated values for all data as a proportion of the ADRB activity of the mean of the 10 malaria-naïve UK serum samples (RLU_n_). To assess inter-assay reproducibility we first considered the African samples only, since it was difficult to index by the mean of the negative (UK) controls if we wanted to measure the negative control samples as well in the same assay. In order to measure how well this indexing (i.e., using RLU_p_ that divides by positive control max RLU, or using RLU_n_ that uses a negative control) removes the variability due to PMN donor, we partitioned the R square from the model with sample and PMN donor included into two parts: the percent due to PMN donor (%**R**^**2**^_**donor**_) and the percent due to sample (%**R**^**2**^_**sample**_ = 100 - %**R**^**2**^_**donor**_). We additionally considered Log_10_ RLU_p_ and Log_10_ RLU_n_. This analysis showed that %**R**^**2**^_**donor**_ was smaller when indexed against the positive control, as compared to indexing against the UK samples (Table 2). Both RLU_p_ and Log_10_ RLU_p_ performed similarly well (Table 2).

When UK samples (negative controls) were included in the analysis, we generally saw the same trend: the positive control gives lower values of %**R**^**2**^_**donor**_ than either no indexing or indexing by the negative control. As a result, the stock of pooled hyper-immune Kenyan serum was continued as the control against which all further samples were indexed.

In addition to indexing by the positive control, we could additionally repeat the analysis on different PMN donors to reduce the dependence of the results on PMN donor. Increasing the number of PMN donors will increase the reproducibility, but how many are sufficient? To answer this question we performed simulations assuming that the errors due to PMN donor (on the Log_10_ RLU_p_) are normally distributed (this assumption appeared reasonable based on graphical analysis; not included). To compare different numbers of donors, we compared the width of confidence intervals (CIs) based on the t-distribution (which is used for normal data).

The width of the 95% CIs were then compared between assay outputs using different numbers of replicates/donors (*n* = 2, 3, …, 8) by simulating 1000 data sets. There was a substantial decrease in the length of CIs between data simulated with samples evaluated with two donors compared to three (Table 3). Additional replicates had a decreasing impact on the reduction of CI length. Considering the level of donor effect can be reduced by adding further PMN donor replicates, and considering the practicality of conducting the assay with many PMN donors, it was determined that three replicates/donors should be sufficient for most applications.

### ADRB cohort analysis

A standardized protocol for measuring the ADRB activity induced by a sample was thus adopted: plates coated with 100 μL PEMS at 18.5 × 10^5^ schizonts/mL; 100 μL PMNs at 1 × 10^7^ PMNs/mL; un-treated serum diluted 1:50; and samples measured in singlicate with three different PMN donors after which data were indexed against a positive control which was included on each plate and all data being log transformed; the mean of the three indexed and log transformed data points was reported.

**Figure 5:**
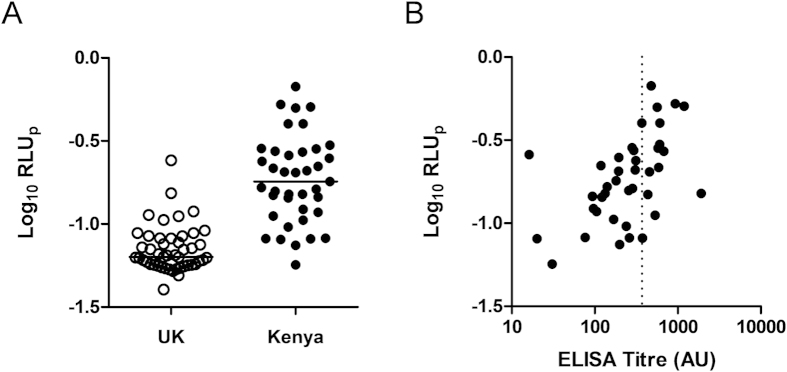
Assessment of ADRB activity in a cohort of Kenyan and UK adults. Serum was collected from adults from both Kenya and the UK. (**A**) ADRB activity induced by sera from UK (open circles *n* = 47) and Kenyan adults (closed symbols; *n* = 39) and is reported as Log_10_ RLU_p_. (**B**) Anti-PEMS total IgG ELISA titer was plotted against Kenyan anti-PEMS ADRB activity. Lines on dot plots represent medians. Dotted line indicates mean ELISA response (370 AU). UK control sera were reported as negative by this ELISA (not shown).

Using this protocol, a cohort of serum samples, separate from the one used in the studies above, from 39 Kenyan adults and 47 UK malaria-naïve adults was assessed. Despite overlap between the malaria-naïve and malaria-exposed groups, sera from the Kenyan adults induced significantly higher ADRB activity than sera from UK volunteers (*P* < 0.0001; Fig. 5A). We also assessed whether ADRB activity correlated with PEMS-specific ELISA titer. When the Kenyan samples were plotted there was a significant but relatively weak relationship (*r*_*s*_ = 0.57, *P* = 0.0001; Fig. 5B), with samples of mean ELISA response (370 AU) showing a high variation – from maximal to minimal ADRB activity as measured in this cohort. We also tested a subset of these sera using the alternative protein-based assay methodology using recombinant PfMSP1 antigen (Supplementary Figure 5). These data showed a strong and highly significant correlation between ADRB activity and ELISA titer. Overall, these data suggest that the PEMS-based assay, as undertaken here, is suitable for testing for anti-merozoite ADRB activity in the sera of malaria-exposed individuals, and can yield extra information on the functionality of the antibody response that is complementary to those obtained by PEMS-based ELISA.

## Discussion

This study describes an optimized protocol for the ADRB assay to assess antibody reactivity against blood-stage malaria parasites. While protocols for this assay have been previously reported^19^,^20^, this is the first time the ADRB assay has been standardized for reproducibility. We have also used this protocol upon a study cohort of 86 individuals showing that it could easily be applied to the immunological assessment of NAI or vaccine-induced responses from cohorts of individuals enrolled in epidemiological studies or clinical trials.

We were able to show a significant difference between ADRB activity induced by sera from malaria-naïve UK adults and those from malaria-exposed Kenyan adults. However, the data reported here also suggest it is important to include a ‘malaria-naïve’ cohort in all future ADRB studies to define baseline responses, and we would also recommend that at least a single ‘negative’ sample be run on each assay plate as a quality control measure to ensure that background responses are minimized. The low or lack of reactivity of some African sera tested here is also likely a reflection of low titers of antibody, or an absence of antibodies, that can bind to the merozoite surface and elicit functional ROS release from neutrophils. Future studies will seek to formally associate this assay output with clinical outcome in well-characterized cohorts.

When investigating whether any prior processing of samples was required before they could be tested in the assay, we observed that assay output was largely independent of any effects mediated by complement, as confirmed by heat inactivation of serum which did not affect the ability of serum from hyper-immune or malaria-naïve volunteers to induce ADRB activity. We also observed no differences when using serum or plasma from matched samples in a clinical vaccine trial indicating that plasma components (e.g. fibrin) and lithium heparin anticoagulant do not affect ADRB activity. Another recently reported study on this assay identified some change in the PMN response profile when purified IgG or decomplemented plasma was used, as opposed to native plasma^20^. We elected to use native serum/plasma to better reflect the *in vivo* situation. While the involvement of complement proteins in PMN activation and ROS release has been described^35^,^36^, it seems likely that PMN activation occurs via a complex interplay of immune complex size, epitope density, IgG subclass and complement, signalling via FcR (CD16 and CD32) and complement receptors (CR1 and CR3)^37^. Subsequently, when stimulating PMNs with merozoites and polyclonal sera, immune complexes which do not activate complement pathways may still be able to activate PMNs by alternate pathways (e.g. CD32), making the effect of complement proteins not easy to distinguish. Indeed, the recent report by Kapelski *et al.* identified CD32a as a key determinant of ROS production in this assay^20^. In line with this, when we did look at individual isotypes in a related PfMSP1 protein-based ADRB assay, purified epitope-matched monoclonal antibodies (and thus samples lacking complement) of different isotypes induced similar activation profiles to those previously described^37^ with IgA > IgG3 > IgG1 except at high concentrations. The role of Ig isotypes such as IgA in natural immunity, and in mediation of ADRB activity, remains to be determined, however data from humanized rodent models suggest a limited contribution^32^, and IgA responses in the same Kenyan cohort appeared limited when tested by ELISA against two merozoite antigens^38^.

We next aimed to address a key limitation in the field of cell-based assays: optimising assay reproducibility, and in particular, inter-assay variability. Guidelines for assay validation, as outlined in the International Conference on Harmonisation (ICH) Harmonised Tripartite Guidelines Q2(R1) state the importance of assessing reproducibility for any assay to be widely used in research^39^. The impact of a highly reproducible assay to the field cannot be understated, as shown by the extensive use of standardized ELISAs in the field of malaria vaccine development^40^. It was apparent that a significant source of inter-assay variation in the ADRB assay resulted from using PMNs isolated from different donors and even from the same donor at different times. Plate-to-plate variation, tested by using the same PMN preparation on different plates, was not directly assessed, although the permutation test showed no indication of plate-to-plate variation. Differences in PMN FcR expression profile may account for some of the donor differences, especially given the importance of CD32 in mediating ADRB activity^20^. We observed donors with both CD32^hi^ and CD32^lo^ PMN phenotypes. It is unknown whether these phenotypic differences reflect genetic polymorphisms in CD32 genes (leading to differential expression or differential binding of flow cytometry antibodies), or simply differences in *in vivo* inflammatory environments at the time of phlebotomy resulting in differential CD32 expression^41^. Whether individuals with a CD32^hi^ phenotype are capable of inducing a higher overall ADRB level *in vivo* remains unknown and is not able to be tested without a personalized assay format matching donor serum and PMNs. This could form an avenue of future research, but would require specialized assay set-up with donor screening, whereas the current protocol enables reproducible results without this necessity. Adding support to the argument that differential CD32 expression, or at least differences in CD32 signalling activity, may lead to heightened ADRB levels *in vivo*, are the numerous associations between CD32 polymorphisms and clinical malaria outcome^42^,^43^,^44^,^45^.

In the context of the ADRB assay, where the primary focus is to assess the potential of antibodies to activate neutrophils, we found that despite neutrophil donor phenotype providing a possible source of inter-assay variation, inter-assay variability could be sufficiently reduced by testing each sample with three neutrophil donors. Given that, in many laboratories, one of the limiting factors for high throughput of this assay will be the number of neutrophil donors available, we suggest that excluding certain donors based on their PMN phenotype is not practical, and we were able to attain acceptable reproducibility without doing so.

Having defined the assay parameters, we assessed ADRB activity in a cohort of samples from Kenyan and UK adult volunteers. We also compared these to ELISA titers. When we used a related assay which assessed responses against recombinant protein instead of blood-stage malaria parasites, like the PEMS-based assay, Kenyan sera induced higher activity than UK sera and ADRB activity strongly correlated with PfMSP1 antigen-specific IgG ELISA titers. A similar result was observed with anti-PEMS ADRB activity, when correlated against PEMS-specific IgG ELISA titer. Importantly, however, a complete range of ADRB responses were elicited from samples with an equivalent PEMS-specific IgG ELISA titer of 370AU, suggesting that valuable extra information should be gained by assaying for ADRB activity when seeking to associate immunological parameters with protection and/or clinical outcome. In this case, it appears that the presentation of antigens on the surface of the parasite is important in the induction of functional activity and cannot be assessed holistically with traditional ELISA-based methods. Work involving a different assay in the field of blood-stage malaria, the GIA assay, has also shown the importance of assessing functional activity of human sera instead of ELISA titer alone, especially when considering combinations of antibodies against many antigen specificities and despite the association between IgG titer and GIA activity for some individual antigens when studies in isolation^46^,^47^.

Another recent study on the ADRB assay adds further weight to the importance of antigen type and presentation in the assay, reportedly effecting the cellular location of respiratory burst^20^. A solid-phase assay (as used here) led to external production of ROS, in comparison to using whole merozoites in solution which led to phagocytosis and intracellular ROS production. In agreement with our data, both versions of the assay correlated with anti-schizont lysate ELISA^20^, despite other differences in methodology, such as the use of cell-permeable luminol in comparison to the less lipophilic isoluminol used here and in the study by Joos *et al.*^19^ for detection of the extracellular ROS. Given the assay using luminol and merozoites in solution allows for detection of merozoite phagocytosis followed by intracellular ROS production^20^, it may be that the ADRB assay in general can be extended in future to distinguish these two possible types of anti-parasitic mechanism – killing of the parasite in the bloodstream via a strong ROS response, versus phagocytosis followed by intracellular killing. However, given the above noted correlations, it may be that both assay formats would give similar readouts irrespective of the actual mechanism of ROS production. This question may warrant further investigation in future studies.

While we have applied the assay method developed here to blood-stage malaria, it could similarly be used to assess antibody induced PMN activation against any antigen or pathogen. The PEMS-specific ADRB assay is not overly labor intensive and can be used to screen large cohorts quickly. Using this protocol, 50 samples are comfortably assayed in a day. The two major limiting factors on even higher throughput in the protocol in our hands were PEMS production, which we overcame by setting up large cultures (up to 150 mL) of parasites for harvesting, and sourcing blood donors for isolation of fresh PMNs. Importantly we show that both serum and plasma can be used in the assay without affecting the result, thus negating the need to source specifically prepared samples from the clinic.

We thus describe a standardized protocol for the ADRB assay, providing a valuable tool for the assessment of NAI and vaccine development. The current paucity in pre-clinical assays available for assessing the function of either vaccine-induced or naturally-acquired antibodies presents a major problem in the study of immunity against blood-stage malaria. Due to this paucity, the field to date has relied on assessing cell-independent anti-merozoite activity using the GIA assay. In more recent years, other cell-based phagocytic assays have also been described^10^,^11^ as well as an assay of Ab-C’ inhibition of merozoite invasion^15^. Consequently, the ADRB assay, as presented here, should complement the use of the GIA neutralization assay and newer phagocytic and complement-based assays in studies of immunity to, and vaccination against, blood-stage malaria.

## Materials and Methods

### Serum sample collection

The use of all serum or plasma samples in this study was in accordance with approvals from the relevant ethical and regulatory bodies as detailed below.

UK adult serum was obtained from healthy malaria-naïve adult volunteers enrolled in either a Phase I (VAC036) or a Phase IIa (VAC039) malaria vaccine clinical trial with appropriate informed consent, and regulatory and ethical approvals, as previously reported and approved by the UK Gene Therapy Advisory Committee, the Berkshire Research Ethics Committee and the UK Medicines and Healthcare products Regulatory Agency^31^,^48^.

Kenyan adult sera were collected during adult cross-sectional surveys between 2006 and 2008 from the villages surrounding the Chonyi area in Kilifi, Kenya that experiences moderate malaria transmission with an EIR of 10–100 infective bites/person/year; these adults are considered to have substantial naturally-acquired immunity as evidenced by the decline in clinical episodes of malaria with age^49^. Scientific and ethical approvals for the Kenyan serum samples were granted by the Kenya National Scientific and Research Ethics Committees, respectively, SSC No. 1131. The samples were kindly provided by Prof Kevin Marsh (KEMRI-Wellcome, Kilifi, Kenya).

Malian plasma samples were collected from volunteers in the village of Kenieroba, Mali in 2009 as part of a cohort study conducted by the National Institute of Allergy and Infectious Diseases (NIAID, NIH, USA: NCT00669084). The approval of the human study was obtained from the Ethical Review Committees of the Faculty of Medicine, Pharmacy, and Odontostomatology at the University of Bamako (Mali) and the NIAID (IRB no. 08-I-N120). Individual written informed consent was obtained from all participants.

Where applicable, serum was heat inactivated by heating to 56 ^o^C for 30 min

### *P. falciparum* parasite preparations

#### P. falciparum

3D7 clone parasites were routinely maintained in culture with RBCs from healthy UK O^+^ donors (ethics approval #06/Q1606/123) and in the presence of pooled human AB serum from healthy UK volunteers (National Blood Service) as previously described^50^. To obtain Parasitophorous vacuolar membrane-Enclosed Merozoite Structures (PEMS), cultures were synchronized with D-sorbitol to lyse mature parasites and enrich ring stages^51^ on two occasions approximately 42 h apart. Approximately 30 h following the second D-sorbitol treatment, late trophozoites and early schizonts were isolated upon a 65% isotonic Percoll density gradient. Infected cells containing late-stage parasites were then cultured for a further 2–8 h with 10 μM Epoxysuccinyl-L-leucylamido(4-guanidino)​butane (E64)^52^,^53^. Once the culture was confirmed to contain >95% fully segmented schizonts by Giemsa staining, infected cells were washed in PBS before dilution to 18.5 × 10^5^ schizonts per mL. The resulting suspension was vortexed vigorously, aliquoted and frozen to rupture the red cell membranes and release merozoites. A single batch of this parasite preparation was made to carry out this entire study and was stored at −20 ^°^C. RBC used in Fig. 1E were prepared using the same protocol upon an uninfected culture of RBC.

### Isolation of polymorphonuclear cells (PMNs)

Human neutrophils/PMNs were isolated from whole blood collected in EDTA vacutainers (BD Biosciences; approval #06/Q1606/123). 4 mL blood was layered upon 5 mL polymorphprep (Axis-Shield Diagnostics Ltd) and centrifuged at 450 × *g* for 40 min. The band containing PMNs was isolated and washed in neutrophil buffer (HBSS (with Ca^2+^ and Mg^2+^), 1% glucose, 0.1% BSA)^54^. Contaminating RBC were lysed with ice cold 0.2% NaCl added for 20 s before the restoration of isotonic conditions by the addition of an equal volume of 1.6% NaCl. Cell viability was confirmed by Trypan blue exclusion and purity determined by Giemsa stained slide before suspension in neutrophil buffer at 1 × 1^7^ PMNs/mL. Cell viability was >99% for all experiments. Giemsa stained slides were examined under a 100x oil immersion objective on a Leica DM2000 microscope and PMN purity assessed in at least 5 fields of view. If PMN purity was <95%, final dilution in neutrophil buffer was adjusted to ensure a final suspension of 1 × 10^7^ PMNs/mL. If PMN purity was below 75% due to RBC contamination, the RBC lysis step was repeated.

### Antibody-dependent respiratory burst (ADRB) assay

100 μL PEMS at 18.5 × 10^5^ schizonts/mL was adsorbed onto Nunc opaque Maxi-sorp 96-well plates (Thermo Scientific) at room temperature (RT) overnight. Plates were then washed three times with PBS and blocked for 1 h with Casein block solution (Pierce, UK) before a second set of 3x washes. 100 μL serum diluted 1:50 in PBS (unless stated otherwise) was then added and incubated for 1 h at 37 ^°^C. Within 2 min of a final wash of the assay plate in PBS, 50 μL isoluminol (Sigma Aldrich, UK) (0.04 mg/mL) and 50 μL isolated human PMNs at 1 × 10^7^ PMNs/mL were added to each well and luminescence was read (in relative light units [RLU]) every 2 min for 1 h using a Varioskan Flash luminometer. For Fig. 1D, serum was heat inactivated by heating to 56 ^°^C for 30 min. A positive control sample of pooled hyper-immune Kenyan serum was tested on each plate at a dilution of 1:50 and used to index samples as described in Results.

### Flow cytometry

9 mL whole blood was collected from healthy volunteers in EDTA vacutainers (BD Biosciences; approval #06/Q1606/123). PMNs were isolated as above and resuspended in 500 μL 0.1% BSA in PBS (PBS/BSA). 150 μL of the resulting cell suspension was surface stained for 30 min at 4 ^o^C with PE-Cy7-labelled anti-CD16 (clone 3G8), FITC-labelled anti-CD32 (clone 3D3) and PerCPCy5.5-labelled anti-CD64 (clone 10.1) (all anti-human antibodies from BioLegend). Cells were then washed twice in 150 μL PBS/BSA and resuspended in 200 μL PBS/BSA. Samples were run on an LSRII flow cytometer (BD Bioscience, USA) with stopping gates set at 1 × 10^6^ total events. Neutrophils were identified as the CD16^+^ granulocyte population (Supplementary Figure 4). Data were analyzed using FlowJo v8.8.7.

### Scanning electron microscopy

The PEMS preparation was allowed to settle onto 13 mm glass coverslips coated with 1% 40 kDa linear polyethylenimine Max (Polysciences Inc.) for 1 h. Supernatant was removed and replaced with primary fixative (2.5% glutaraldehyde in 0.1 M PBS) for incubation overnight at 4 °C before washing with 0.1 M sodium cacodylate buffer (pH 7) and further incubation in 1% tannic acid in 0.1 M sodium cacodylate buffer for 1 h at RT. Samples were washed again for 3 × 10 min in sodium cacodylate buffer, then incubated in secondary fixative (1% osmium tetroxide, 0.1 M sodium cacodylate buffer, pH 7) for 1 h at RT. Samples were rinsed for 2 × 5 min with water, then taken through an ethanol dehydration series as follows: 50% ethanol for 15 min, 70% ethanol overnight at 4 °C, 90% and 95% ethanol for 15 min each and then 100% ethanol for 3 × 30 min. Samples were dried in a Touismis Auto Samdri-815 critical point drier, with a purge time of 15 min. Coverslips were mounted cell side up onto aluminium stubs using carbon tape. Samples were coated with approximately 10 nm gold/palladium in a BioRad E5100 Series II sputter coater, then imaged using the secondary electron detector on a JEOL JSM-6390 at 5 kV, spot size 30, working distance 5 mm.

### Enzyme Linked Immunosorbent Assay (ELISA)

Total IgG ELISAs were carried out using a standardized ELISA methodology^40^, as previously described^55^. Briefly, plates were coated with PEMS at 18.5 × 10^5^ schizonts/mL. Arbitrary units (AU) were determined by comparison to a standard curve of pooled hyper-immune sera from Kenyan adults. The AU are defined by the reciprocal of the dilution of this reference serum which gives an optical density readout of 1.0. The optical density was read at 405 nm (OD_405_) using a BioTek EL 800 Microplate Reader (BioTek, UK).

### Statistical analysis

Comparisons between two independent groups were conducted using Mann-Whitney tests, or Wilcoxon signed rank test when data were paired. Correlations between ELISA titer and ADRB activity were tested using Spearman’s rank correlation (r_s_). We tested for PMN donor effects using a linear regression on Log_10_ max RLU, using a likelihood ratio test comparing the model with main effects for sample and plate to the model that additionally has PMN donor effects. We repeated that test after adjusting for day instead of plate. To test for a plate effect we could not use similar methods since each PMN donor was tested on only one plate. Instead, we performed a permutation test. Specifically, we permuted the second PMN donor on each plate, and for each permutation fit the linear regression of Log_10_ max RLU by sample and plate, ordered the 24 permutations by the F-statistic of the “plate” effects, and the *P*-value is the proportion of all 24 F-statistics that are greater than or equal to the original.

Interclass correlation coefficient (ICC) analysis^34^ was used to assess the repeatability of measurements under the same conditions in the cases in which samples were tested in duplicate. To determine the effect of PMN donor, a linear regression (using sample and PMN donor as main effects) on each response was run, and the total proportion of the variance explained by the model (*R*^*2*^), was partitioned into *R*^*2*^ due to sample (*R*^*2*^_*s*_), *R*^*2*^ due to donor (*R*^*2*^_*d*_) using hierarchical partitioning^56^. Confidence intervals (CI) were calculated using a non-parametric bootstrap with the percentile intervals using 2000 replications^57^. Table 2 depends on the assumption of normality of the residuals from the linear model with only sample included. This assumption was checked graphically using kernel density estimation. For comparing the singlicates versus duplicates we compared R^2^ values from the model with sample only included for the two methods. We measured R^2^_s Dup._ with the response equal to the average of the replicates. We measured R^2^_s Sing._ by including both replicates and calculating the R^2^ in the usual way as data. This is like averaging the R^2^_s Sing._ values from repeatedly randomly selecting one replicate. Statistical analyses for Figures 1,3 and 5, and Supplementary Figures 2 and 5 were carried out using Prism v.5.03 (GraphPad, USA) and other statistical tests were performed in R (version 2.15.2) and two-sided *P*-values  < 0.05 were considered significant.

## Additional Information

**How to cite this article**: Llewellyn, D. *et al.* Standardization of the antibody-dependent respiratory burst assay with human neutrophils and *Plasmodium falciparum* malaria. *Sci. Rep.* **5**, 14081; doi: 10.1038/srep14081 (2015).

## Supplementary Material

Supplementary Information

## Figures and Tables

**Table 1:** Singlicates versus duplicates.

	**African**			**African + UK**		
	**Singlicates**	**Duplicates**	%Increase	**Singlicates**	**Duplicates**	%Increase
RLU	0.622	0.635	2.14	0.734	0.745	1.45
Log_10_ RLU	0.690	0.704	2.04	0.734	0.744	1.36
RLU_p_	0.787	0.840	6.68	0.860	0.893	3.81
Log_10_ RLU_p_	0.888	0.913	2.88	0.847	0.863	1.85

Comparison of *R*^2^ from the linear models explained by sample effects only 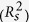 for samples measured in either singlicate or duplicate wells using either: malaria-endemic African samples only (African), or both malaria-endemic African and malaria-naïve UK samples combined (African + UK). The percentage increase in 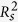 between testing samples in duplicate and testing singlicates is also shown (as calculated in the text). RLU_p_ is the ADRB activity in RLU of a test sample divided by the ADRB activity in RLU elicited by a positive control of pooled hyper-immune Kenyan adult serum. Analyses done with results in RLU and RLU_p_ which were Log transformed are also shown.

**Table 2:** Minimizing variation due to donor by indexing.

AssayReadout	**African (% R^2^_donor_)**	**African + UK (%R^2^_donor_)**
RLU	24.5 (17.8–33.8)	12.5 (9.5–17.6)
Log_10_ RLU	25.9 (18.7–34.9)	19.4 (15.7–24.0)
RLU_p_	3.5 (2.4–10.2)	1.4 (1.2–4.6)
Log_10_ RLU_p_	2.0 (1.7–7.4)	5.3 (3.6–9.5)
RLU_n_	44.3 (35.3–52.1)	18.7 (13.8–24.9)
Log_10_ RLU_n_	27.8 (21.6–35.6)	6.8 (4.8–10.9)

ADRB activity against PEMS was determined for 13 malaria-endemic African and 10 malaria-naïve UK serum or plasma samples using 8 different neutrophil donors. The Table reports percentage of assay output *R*^*2*^ explained by donor effects on African samples only (African), or both African and UK (African + UK) samples after no indexing (RLU); indexing against a positive hyper-immune Kenyan pool (RLU_p_); indexing against the mean of the 10 malaria-naïve UK samples (RLU_n_); and log transformation of data. Parentheses indicate 95% CIs.

**Table 3:** Number of donor replicates.

**Number Donors**	**95% CI length (% n = 2)**
2	100.0
3	30.4
4	20.3
5	16.1
6	13.9
7	12.3
8	11.2

Table shows the mean length of the 95% CI using different numbers of donors, as a percentage of the length of the 95% CI calculated using only two donors. These calculations are based on the normality assumption (deemed reasonable by graphical methods, not shown), and were done by simulation using 1000 replications.

## References

[b1] The malERA Consultative Group on Vaccines. A Research Agenda for Malaria Eradication: Vaccines. PLoS Med 8, e1000398 (2011). 10.1371/journal.pmed.1000398 PMC3026701 21311586

[b2] Murray C. J. L. *et al.* Global malaria mortality between 1980 and 2010: a systematic analysis. Lancet 379, 413–431 (2012). 22305225 10.1016/S0140-6736(12)60034-8

[b3] World Health Organisation. World Malaria Report 2013. (Geneva, 2013).

[b4] Langhorne J., Ndungu F. M., Sponaas A.-M. & Marsh K. Immunity to malaria: more questions than answers. Nat Imm 9, 725–732 (2008). 10.1038/ni.f.205 18563083

[b5] Wright G. J. & Rayner J. C. *Plasmodium falciparum* erythrocyte invasion: combining function with immune evasion. PLoS Path 10, e1003943 (2014). 10.1371/journal.ppat.1003943 PMC3961354 24651270

[b6] Goodman A. L. & Draper S. J. Blood-stage malaria vaccines - recent progress and future challenges. Ann Trop Med Parasitol 104, 189–211 (2010). 20507694 10.1179/136485910X12647085215534

[b7] Halbroth B. R. & Draper S. J. Recent developments in malaria vaccinology. Adv Parasitol 88, 1–49 (2015). 25911364 10.1016/bs.apar.2015.03.001

[b8] Duncan C. J. A. *et al.* Impact on malaria parasite multiplication rates in infected volunteers of the protein-in-adjuvant vaccine AMA1-C1/Alhydrogel+CPG 7909. PLoS ONE 6, e22271 (2011). 21799809 10.1371/journal.pone.0022271 PMC3142129

[b9] Duncan C., Hill A. V. S. & Ellis R. Can growth inhibition assays (GIA) predict blood-stage malaria vaccine efficacy? Hum Vaccin Immunother 8, 706–714 (2012). 22508415 10.4161/hv.19712 PMC3495712

[b10] Hill D. L. *et al.* Efficient measurement of opsonising antibodies to *Plasmodium falciparum* merozoites. PLoS ONE 7, e51692 (2012). 23300556 10.1371/journal.pone.0051692 PMC3530572

[b11] Osier F. H. *et al.* Opsonic phagocytosis of *Plasmodium falciparum* merozoites: mechanism in human immunity and a correlate of protection against malaria. BMC Medicine 12, 108 (2014). 24980799 10.1186/1741-7015-12-108 PMC4098671

[b12] Turrini F. *et al.* Phagocytosis of *Plasmodium falciparum*-infected human red blood cells by human monocytes: involvement of immune and nonimmune determinants and dependence on parasite developmental stage. Blood 80, 801–808 (1992). 1638029

[b13] Celada A., Cruchaud A. & Perrin L. H. Phagocytosis of *Plasmodium falciparum*-parasitized erythrocytes by human polymorphonuclear leukocytes. J Parasitol 69, 49–53 (1983). 6338199

[b14] Chan C. L., Rénia L. & Tan K. S. W. A simplified, sensitive phagocytic assay for malaria cultures facilitated by flow cytometry of differentially-stained cell populations. PLoS ONE 7, e38523 (2012). 22675573 10.1371/journal.pone.0038523 PMC3366917

[b15] Boyle M. J. *et al.* Human antibodies fix complement to inhibit *Plasmodium falciparum* invasion of erythrocytes and are associated with protection against malaria. Immunity 42, 580–590 (2015). 25786180 10.1016/j.immuni.2015.02.012 PMC4372259

[b16] Lunel F. & Druilhe P. Effector cells involved in nonspecific and antibody-dependent mechanisms directed against *Plasmodium falciparum* blood stages *in vitro*. Infect Immun 57, 2043–2049 (1989). 2659533 10.1128/iai.57.7.2043-2049.1989 PMC313839

[b17] Bouharoun-Tayoun H., Attanath P., Sabchareon A., Chongsuphajaisiddhi T. & Druilhe P. Antibodies that protect humans against *Plasmodium falciparum* blood stages do not on their own inhibit parasite growth and invasion *in vitro*, but act in cooperation with monocytes. J Exp Med 172, 1633–1641 (1990). 2258697 10.1084/jem.172.6.1633 PMC2188756

[b18] Jepsen M. P. G. *et al.* The malaria vaccine candidate GMZ2 elicits functional antibodies in individuals from malaria endemic and non-endemic areas. J Infect Dis 208, 479–488 (2013). 23624363 10.1093/infdis/jit185

[b19] Joos C. *et al.* Clinical protection from *falciparum* malaria correlates with neutrophil respiratory bursts induced by merozoites opsonized with human serum antibodies. PLoS ONE 5, e9871 (2010). 20360847 10.1371/journal.pone.0009871 PMC2845614

[b20] Kapelski S., Klockenbring T., Fischer R., Barth S. & Fendel R. Assessment of the neutrophilic antibody-dependent respiratory burst (ADRB) response to *Plasmodium falciparum*. *J Leuk* Biol 96, 1–12 (2014). 10.1189/jlb.4A0614-283RR PMC4226792 25118179

[b21] Pleass R. J. *et al.* Novel antimalarial antibodies highlight the importance of the antibody Fc region in mediating protection. Blood 102, 4424–4430 (2003). 12855589 10.1182/blood-2003-02-0583

[b22] Kumaratilake L. M. & Ferrante A. Opsonization and phagocytosis of *Plasmodium falciparum* merozoites measured by flow cytometry. Clin Diagn Lab Immunol 7, 9–13 (2000). 10618269 10.1128/cdli.7.1.9-13.2000 PMC95814

[b23] Sun T. & Chakrabarti C. Schizonts, merozoites, and phagocytosis in *falciparum* malaria. Ann Clin Lab Sci 15, 465–469 (1985). 3904592

[b24] Llewellyn D. *et al.* Assessment of antibody-dependent respiratory burst activity from mouse neutrophils on *Plasmodium yoelii* malaria challenge outcome. J Leuk Biol 95, 369–382 (2014). 10.1189/jlb.0513274 PMC3896657 24163420

[b25] Clark I. A. & Hunt N. H. Evidence for reactive oxygen intermediates causing hemolysis and parasite death in malaria. Infect Immun 39, 1–6 (1983). 6822409 10.1128/iai.39.1.1-6.1983 PMC347899

[b26] Murray H. Susceptibility of leishmania to oxygen intermediates and killing by normal macrophages. J Exp Med 153, 1302–1315 (1981). 7252418 10.1084/jem.153.5.1302 PMC2186145

[b27] Murray H. W. & Cohn Z. A. Macrophage oxygen-dependent antimicrobial activity. I. Susceptibility of *Toxoplasma gondii* to oxygen intermediates. J Exp Med 150, 938–949 (1979). 92521 10.1084/jem.150.4.938 PMC2185675

[b28] Friedman M. J. Oxidant damage mediates variant red cell resistance to malaria. Nature 280, 245–247 (1979). 377105 10.1038/280245a0

[b29] Greve B. *et al.* High oxygen radical production is associated with fast parasite clearance in children with *Plasmodium falciparum* malaria. J Infect Dis 179, 1584–1586 (1999). 10228089 10.1086/314780

[b30] Sanni L. A. *et al.* Are reactive oxygen species involved in the pathogenesis of murine cerebral malaria? J Infect Dis 179, 217–222 (1999). 9841842 10.1086/314552

[b31] Sheehy S. H. *et al.* ChAd63-MVA–vectored blood-stage malaria vaccines targeting MSP1 and AMA1: assessment of efficacy against mosquito bite challenge in humans. Mol Ther 20, 2355–2368 (2012). 23089736 10.1038/mt.2012.223 PMC3519995

[b32] Shi J. *et al.* The generation and evaluation of recombinant human IgA specific for *Plasmodium falciparum* merozoite surface protein 1-19 (PfMSP1_19_). BMC biotechnol 11, 77–94 (2011). 21781305 10.1186/1472-6750-11-77 PMC3199766

[b33] McIntosh R. S. *et al.* The importance of human FcgRI in mediating protection to malaria. PLoS Path 3, e72 (2007). 10.1371/journal.ppat.0030072 PMC1868954 17511516

[b34] Koch G. G. Intraclass Correlation Coefficient. in Encyclopedia of Statistics, Vol. 4 (eds. Kotz S. & Johnson N. L.) (Wiley, New York, 1983).

[b35] Camous L. *et al.* Complement alternative pathway acts as a positive feedback amplification of neutrophil activation. Blood 117, 1340–1349 (2011). 21063021 10.1182/blood-2010-05-283564

[b36] Morgan B. P. Complement membrane attack on nucleated cells: resistance, recovery and non lethal effects. Biochem J 264, 1–14 (1989). 2690818 10.1042/bj2640001 PMC1133540

[b37] Voice J. K. & Lachmann P. J. Neutrophil Fcγ and complement receptors involved in binding soluble IgG immune complexes and in specific granule release induced by soluble IgG immune complexes. Eur J Immunol 27, 2514–2523 (1997). 9368604 10.1002/eji.1830271008

[b38] Biswas S. *et al.* Assessment of humoral immune responses to blood-stage malaria antigens following ChAd63-MVA immunization, controlled human malaria infection and natural exposure. PLoS One 9, e107903 (2014). 25254500 10.1371/journal.pone.0107903 PMC4177865

[b39] International Conference on Harmonization of Technical Requirements for Registration of Pharmaceuticals for Human Use (ICH). Validation of analytical procedures: text and methodology Q2(R1). Vol. 2014 (ICH Official web site http://www.ich.org, 2005). Accessed 1^st^ May 2015.

[b40] Miura K. *et al.* Development and characterization of a standardized ELISA including a reference serum on each plate to detect antibodies induced by experimental malaria vaccines. Vaccine 26, 193–200 (2008). 18054414 10.1016/j.vaccine.2007.10.064 PMC2253722

[b41] Pan L., Kreisle R. & Shi Y. Detection of Fcg receptors on human endothelial cells stimulated with cytokines tumour necrosis factor-alpha (TNF-a) and interferon-gamma (IFN-g). Clin Exp Immunol 112, 533–538 (1998). 9649226 10.1046/j.1365-2249.1998.00597.x PMC1904983

[b42] Omi K. *et al.* Fcγ receptor IIA and IIIB polymorphisms are associated with susceptibility to cerebral malaria. Parasitol Int 51, 361–366 (2002). 12421634 10.1016/s1383-5769(02)00040-5

[b43] Cooke G. S. *et al.* Association of Fcg receptor Ila (CD32) polymorphism with severe malaria in West Africa. Am J Trop Med Hyg 69, 565–568 (2003). 14740869

[b44] Shi Y. P. *et al.* Fcγ receptor IIa (CD32) polymorphism is associated with protection of infants against high-density *Plasmodium falciparum* infection. VII. Asembo Bay Cohort Project. J Infect Dis 184, 107–111 (2001). 11398118 10.1086/320999

[b45] Ouma C. *et al.* Functional haplotypes of Fc gamma (Fcγ) receptor (FcγRIIA and FcγRIIIB) predict risk to repeated episodes of severe malarial anemia and mortality in Kenyan children. Hum Genet, 131, 289–299 (2012). 21818580 10.1007/s00439-011-1076-8 PMC3258363

[b46] Polhemus M. E. *et al.* Phase I dose escalation safety and immunogenicity trial of *Plasmodium falciparum* apical membrane protein (AMA-1) FMP2.1, adjuvanted with AS02A, in malaria-naïve adults at the Walter Reed Army Institute of Research. Vaccine 25, 4203–4212 (2007). 17442466 10.1016/j.vaccine.2007.03.012

[b47] Aoki S. *et al.* Serine repeat antigen (SERA5) is predominantly expressed among the SERA multigene family of *Plasmodium falciparum*, and the acquired antibody titers correlate with serum inhibition of the parasite growth. J Biol Chem 277, 47533–47540 (2002). 12244052 10.1074/jbc.M207145200

[b48] Sheehy S. H. *et al.* Phase Ia clinical evaluation of the safety and immunogenicity of the *Plasmodium falciparum* blood-stage antigen AMA1 in ChAd63 and MVA vaccine vectors. PLoS One 7, e31208 (2012). 22363582 10.1371/journal.pone.0031208 PMC3283618

[b49] Marsh K. & Kinyanjui S. Immune effector mechanisms in malaria. Parasite Immunol 28, 51–60 (2006). 16438676 10.1111/j.1365-3024.2006.00808.x

[b50] Williams A. R. *et al.* Enhancing blockade of *Plasmodium falciparum* erythrocyte invasion: assessing combinations of antibodies against PfRH5 and other merozoite antigens. PLoS Path 8, e1002991 (2012). 10.1371/journal.ppat.1002991 PMC3493472 23144611

[b51] Lambros C. & Vanderberg J. P. Synchronization of *Plasmodium falciparum* erythrocytic stages in culture. J Parasitol 65, 418–420 (1979). 383936

[b52] Rosenthal P. J., Kim K., McKerrow J. H. & Leech J. H. Identification of three stage-specific proteinases of *Plasmodium falciparum*. J Exp Med 166, 816–821 (1987). 3305763 10.1084/jem.166.3.816 PMC2188684

[b53] Salmon B. L., Oksman A. & Goldberg D. E. Malaria parasite exit from the host erythrocyte: A two-step process requiring extraerythrocytic proteolysis. PNAS 98, 271–276 (2001). 11114161 10.1073/pnas.011413198 PMC14580

[b54] Siemsen D. W., Schepetkin I. A., Kirpotina L. N., Lei B. & Quinn M. T. Neutrophil isolation from nonhuman species. in Neutrophil Methods and Protocols, Vol. 412 (eds. Quinn M. T., DeLeo F. R. & Bokoch G. M.) 21–34 (Humana Press, Totowa, 2007). 10.1007/978-1-59745-467-4_3 18453103

[b55] Sheehy S. H. *et al.* Phase Ia clinical evaluation of the *Plasmodium falciparum* blood-stage antigen MSP1 in ChAd63 and MVA vaccine vectors. Mol Ther 19, 2269–2276 (2011). 21862998 10.1038/mt.2011.176 PMC3242658

[b56] Chevan A. & Sutherland M. Hierarchical partitioning. Am Stat 45, 90–96 (1991).

[b57] Efron B. & Tibshirani R. An introduction to the bootstrap, (CRC press, New York, 1993).

