# Supplementary material for: Standardization of the antibody-dependent respiratory burst assay with human neutrophils and Plasmodium falciparum malaria
Source: Sci Rep. 2015 Sep 16;5:14081. doi: 10.1038/srep14081 (PMC4571651; doi:10.1038/srep14081)
Supplement: Supplementary Information [file srep14081-s1.doc]

# Standardization of the antibody-dependent respiratory burst assay with human neutrophils and *Plasmodium falciparum* malaria

David Llewellyn, Kazutoyo Miura, Michael P. Fay, Andrew R. Williams, Linda M. Murungi, Jianguo Shi, Susanne H. Hodgson, Alexander D. Douglas, Faith H. Osier, Rick M. Fairhurst, Mahamadou Diakite, Richard J. Pleass, Carole A. Long & Simon J. Draper.

## Supplementary Material

**Supplementary Figure 1**

Typical luminescence trace from an ADRB inducing sample (solid line) and a UK naïve sample (dotted line) for the ADRB assay.

**Supplementary Figure 2**

ADRB activity in RLUp (defined in Section “Inter-assay reproducibility”) induced on a plate coated with 10 µg/mL GST-PfMSP119 by sera (light) and plasma (dark) diluted 1:50 from volunteers both before (Day 0) and after (Day C-1) ChAd63-MVA PfMSP1 immunization. Bars represent the mean of two assay replicates.

**Supplementary Figure 3**

ADRB activity in RLUp (defined in Section “Inter-assay reproducibility”) elicited on a plate coated with 10 µg/mL GST-PfMSP119 by chimeric human epitope-matched IgG1 (black), IgG3 (dark grey) and IgA (light grey) mAbs against the C1 and E9 epitopes of PfMSP119 at a range of concentrations.

**Supplementary Figure 4**

Whole blood was taken from healthy UK volunteers, and PMNs isolated on polymorphprep gradients. Cells were surface stained with anti-CD16, anti-CD32, and anti-CD64. One million events were acquired in total. **(A)** Singlicates were gated by forward scatter-area (FSC-A) and -height (FSC-H) profiles. **(B)** Granulocytes were then gated by FSC-A and side scatter-area (SSC-A) profiles. **(C)** PMNs were then defined as the CD16+ population.

**Supplementary Figure 5**

Serum was collected from adults from both Kenya and the UK. Each sample was measured in singlicate with three independent PMN donors. **(A)** ADRB activity induced on a GST-PfMSP119 coated plate by sera diluted 1:50 from UK (open circles *n* = 25) and Kenyan adults (closed symbols; *n* = 40). **(B)** Anti-PfMSP119­ total IgG ELISA titer was plotted against Kenyan anti-PfMSP119 ADRB activity (*rs* =0.92, *P* < 0.0001). UK control sera were reported as negative by this ELISA (not shown). Lines on dot plots represent medians.

## Supplementary Methods:

**Serum**

UK adult serum and plasma samples (pre- and post-vaccination) were obtained from healthy malaria-naïve adult volunteers receiving immunization with ChAd63-MVA viral vectored vaccines encoding *P. falciparum* MSP1 (PfMSP1). These volunteers were enrolled in a Phase I/IIa malaria vaccine clinical trial (VAC039) with appropriate informed consent, and regulatory and ethical approvals, as previously reported . Sera were tested pre-immunization (Day 0) and following ChAd63-MVA PfMSP1 immunization but the day before controlled human malaria infection (Day C-1).

**Monoclonal antibody (mAb) production**

Human IgG1 and IgA mAbs recognizing the C1 and E9 epitopes of the 19 kDa C-terminus of *P. falciparum* MSP1 (PfMSP119) were expressed using Chinese hamster ovary (CHO-K1) and HEK-293 cells respectively, as previously described. To generate IgG3 mAbs recognizing the same epitopes, the γ3 constant region was generated by cutting lambdaEZZgamma3 (ATCC® 65691™) using primer pairs (F: 5’-CCT GGA TCC TCG TGG ATA GAC AAG-3’, R: 5’-GAA GAT ATC GAG TTA CTC AGA TCT GGG-3’) and (F: 5’-CTC AGC TCA GAC ACC TTC TC-3’, R: 5’-TCC CTG ATA TCA CTC AGG CCT CAG ACT-3’) and subcloning the resultant constructs into pBluescript II KS(+) using BamHI/EcoRV, and BglII/EcoRV respectively. The thus generated combined γ3 construct was subcloned into pG1D105 (a kind gift from Dr Tarran Jones) with BamHI/EcoRV, then to pCR2.1Topo (Life Technologies) with BamHI/AfeI before finally replacing the γ1 fragment in the pVHexpress plasmid generated for IgG1 expression using BamHI/XbaI. CHO-K1 cells were then co-transfected for relevant heavy and light chain fragments as described previously for IgG1 .

**Protein ADRB assay**

100 µL recombinant GST-PfMSP119 (ETSR allele) fusion protein at 10 µg/mL produced in an *Escherichia coli* expression system as previously described was adsorbed onto Nunc opaque Maxi-sorp 96-well plates (Thermo Scientific) at room temperature (RT) overnight. Plates were then washed three times with PBS and blocked for 1 h with Casein block solution (Pierce, UK) before a second set of 3x washes. 100 µL serum (or plasma where specified) diluted 1:50 in PBS (unless stated otherwise), or an epitope matched anti-PfMSP119 human IgG1, IgG3 or IgA mAb at stated concentrations, was then added and incubated for 1 h at 37oC. Within 2 min of a final wash of the assay plate in PBS, 50µL isoluminol (Sigma Aldrich, UK) (0.04 mg/mL) and 50µL of isolated human PMNs at 1 x 10^7^ PMNs/mL were added to each well and luminescence was read every 2 min for 1 h using a Varioskan Flash luminometer. Maximum RLU over the course of the hour was recorded and indexed against the same hyper-immune pool of serum used in the PEMS-based assay.

**PfMSP119 ELISA**

Total PfMSP119 IgG ELISAs were carried out using a standardized ELISA methodology , as previously described for these clinical trial samples . Briefly, plates were coated with 2 µg/mL GST-PfMSP119 (ETSR allele) fusion protein and antibody units (AU) determined by comparison to a standard curve of a single hyper-immune Kenyan adult. The optical density was read at 405nm (OD405) using a BioTek EL 800 Microplate Reader (BioTek, UK).
